# Towards a More Critical Public Health Understanding of Vaccine Hesitancy: Key Insights from a Decade of Research

**DOI:** 10.3390/vaccines11071155

**Published:** 2023-06-26

**Authors:** Sara Cooper, Charles S. Wiysonge

**Affiliations:** 1 Cochrane South Africa, South African Medical Research Council, Cape Town 7505, South Africa; charles.wiysonge@mrc.ac.za; 2 School of Public Health and Family Medicine, Faculty of Health Sciences, University of Cape Town, Cape Town 7935, South Africa; 3 Department of Global Health, Faculty of Medicine and Health Sciences, Stellenbosch University, Cape Town 7505, South Africa; 4 HIV and Other Infectious Diseases Research Unit, South African Medical Research Council, Durban 4091, South Africa; 5 Vaccine Preventable Diseases Programme, Universal Health Coverage/Communicable and Non-Communicable Diseases Cluster, World Health Organization Regional Office for Africa, Brazzaville P.O. Box 06, Congo

**Keywords:** vaccine hesitancy, global, dominant assumptions, critical public health, qualitative research, social sciences

## Abstract

Vaccine hesitancy has gained renewed attention as an important public health concern worldwide. Against this backdrop, over the last decade, we have conducted various qualitative, social science studies with the broad shared aim of better understanding this complex phenomenon. This has included various Cochrane systematic reviews of qualitative research globally, systematic reviews of qualitative research in Africa, and primary research studies in South Africa. These studies have also explored vaccine hesitancy for various vaccines, including routine childhood vaccination, HPV vaccination and other routine vaccinations for adolescents, and, most recently, COVID-19 vaccination. In this reflective and critical commentary piece we reflect on seven key overarching insights we feel we have gained about this complex phenomenon from the varying studies we have conducted over the past decade. These insights comprise the following: (1) the relationship between vaccine knowledge and hesitancy is complex and may operate in multiple directions; (2) vaccine hesitancy is driven by multiple socio-political forces; (3) vaccine hesitancy may be many things, rather than a single phenomenon; (4) vaccine hesitancy may be an ongoing ‘process’, rather than a fixed ‘stance’; (5) vaccine hesitancy may sometimes be about a ‘striving’, rather than a ‘resisting’; (6) ‘distrust’ as a driver of vaccine hesitancy needs to be better contextualized and disaggregated; and (7) the ‘demand-side’ versus ‘supply/access-side’ distinction of the drivers of suboptimal vaccination may be misleading and unhelpful. In unpacking these insights, we problematize some of the common assumptions within the vaccine hesitancy literature and flag topics that we think could benefit from further scrutiny and debate. Our hope is that this can provide a platform for further engagement on these issues and ultimately contribute towards fostering a more critical public health understanding of vaccine hesitancy.

## 1. Introduction

Vaccine hesitancy has gained renewed attention as an important global public health concern. Most certainly, this is not a new phenomenon—public skepticism and controversies surrounding vaccination are as old as vaccines themselves. However, vaccine hesitancy trends appear to have escalated in scope and scale in recent years [1,2]. Present-day debates around vaccination are increasingly complex, as more vaccines and combinations of vaccines have been introduced into routine immunization programs [3]. Moreover, the modes and speed of global information exchange have been greatly enhanced through the internet and social media [4]. This has led to more rapid sharing of public concerns, false beliefs, and conspiracy theories about vaccines [5,6]. As a potential consequence of these new dynamics, national governments, international organizations, and the research community are repeatedly being confronted with certain individuals or communities who are questioning vaccines, seeking alternative vaccination schedules, and delaying or refusing vaccination in both high-income countries (HICs) and low- and middle-income countries (LMICs) [4,7,8]. 

In response to this situation, in 2011, the World Health Organization (WHO) listed vaccine hesitancy as a priority topic for its Strategic Advisory Group of Experts on Immunization (SAGE) [9], and identified vaccine hesitancy as one of the ten main threats to global health eight years later [10]. Over this time, various international vaccine hesitancy working groups were also established: a SAGE working group on vaccine hesitancy was formed in 2012 [11]; a working group on vaccine confidence was established in the National Vaccines Advisory Committee in the USA in 2013 [12]; a working group on vaccine demand was initiated in 2015 by the United Nations Children Fund (UNICEF) and WHO [13]; a working group on measuring behavioral and social drivers (BeSD) of vaccination was formed by the WHO in 2018 [14]; and a BeSD on COVID-19 vaccination was formed in 2020 [15]. With the recent global outbreaks of diseases such as measles and diphtheria [16,17], as well as the COVID-19 pandemic for which high rates of COVID-19 vaccine hesitancy were identified [18,19], vaccine hesitancy has been placed firmly on the global public health agenda [2]. 

Against this backdrop, over the last decade, we have conducted several qualitative social science studies with the broadly shared aim of understanding vaccine hesitancy better, including what it is, what drives it, and how it might be addressed. Specifically, we have conducted various Cochrane systematic reviews of qualitative research on the nature and drivers of vaccine hesitancy globally for routine childhood vaccines [20] human papillomavirus (HPV) vaccination for adolescents [21], and COVID-19 [22]. We have also conducted two systematic reviews of qualitative research in Africa, with one focusing on vaccine hesitancy for routine childhood vaccines [23] and the other on HPV vaccination for adolescents [24]. Finally, we have conducted various primary, qualitative research studies in South Africa where we have examined vaccine hesitancy for routine childhood vaccines [25] HPV vaccination and other routine vaccinations for adolescents [26,27] and COVID-19 [28]. 

In this reflective and critical commentary piece, we reflect on seven key overarching insights we feel we have gained about this complex phenomenon. We derived these insights by critically, contemplatively, and more subjectively reflecting on our 10 years of research on this topic and associated deep immersion in the subject. These insights cut across the varying studies we have conducted, and we believe have relevance across different vaccines and settings. In reflecting on these insights, we problematize some of the common assumptions within the vaccine hesitancy literature and flag topics which we think could benefit from further scrutiny and debate. Our hope is that this can provide a platform for further engagement on these issues, and ultimately contribute towards fostering a more critical public health understanding of vaccine hesitancy. 

## 2. Results: Overarching Reflective Insights

### 2.1. The Relationship between Vaccine Knowledge and Hesitancy Is Complex and May Operate in Multiple Directions

One of the most persistent assumptions in the vaccine hesitancy literature is that it is driven by limited or a lack of biomedical information (‘knowledge-deficit’ approaches) [29,30,31]. That is, it is assumed that having sufficient medical facts about the benefits and value of vaccines will lead to greater acceptance of them. However, our research has revealed that knowledge about vaccination influences acceptance of it in complex, diverse, and often unexpected ways. 

For example, in our systematic review of the factors that influence parents’ views and practices around routine childhood vaccination in Africa, we found that for some, their hesitancy towards vaccination was driven by inadequate knowledge about vaccines and what they do [23]. However, in our global systematic review on acceptance of routine childhood vaccination, we observed that others who are hesitant towards vaccines have very sophisticated understandings of vaccines, including their composition, functioning, and potential risks [20]. Our global systematic reviews on routine childhood vaccinations [20] and HPV vaccination acceptance [21] also revealed that for some, inadequate knowledge about vaccination had no impact on their attitudes or behaviors towards it; they accepted and received vaccination despite knowing very little about it. In both these reviews, we found that for many others, their lack or scarcity of biomedical knowledge about vaccination actually enhanced their acceptance of it [20,21]. That is, many held beliefs about vaccination and its benefits that were incongruent with, or even contradicted, biomedical understandings of health, disease, and immunity. However, these beliefs served as strong drivers of vaccination acceptance, such as in the case of parents who thought the HPV vaccine protects against HIV/AIDS [21,24] or that routine childhood vaccines may cure various diseases such as measles [20]. 

In addition to challenging the common assumption of a straightforward and positive association between vaccination knowledge and acceptance, the complexity of this relationship revealed through our research also raises questions about what we should be trying to achieve through our public health interventions. That is, should we be seeking to raise awareness about, and compliance with, vaccination? Or is building science literacy so people can make informed decisions and consent for vaccination important? These different goals are likely to give rise to different types of interventions and potential outcomes, which are not necessarily aligned with reducing vaccine hesitancy and increasing vaccine acceptance. For example, providing people with biomedical knowledge about vaccination might dispel certain myths (e.g., that HPV or measles vaccination protects against HIV/AIDS or cures measles, respectively), and yet this might also remove a key motivator for vaccination and in turn reduce acceptance of it. Similarly, improving the science literacy of those who receive vaccination despite knowing very little about it might enhance their capacity to make informed decisions and give informed consent, and yet this could also increase their hesitancy towards vaccination. Ultimately, these potential tensions around the objectives of our public health interventions require more critical consideration. 

### 2.2. Vaccine Hesitancy Is Driven by Multiple Socio-Political Forces

A central finding across our research globally, regionally, and in South Africa has been that vaccine hesitancy is driven by more complex social and political factors beyond the individual. For example, we have found that vaccine hesitancy may be influenced by people’s broader worldviews about health and illness; by the vaccination ideas and practices of people’s social networks and communities; by wider political issues and relations of power, and particularly the impact these have on peoples’ trust (or distrust) in those associated with vaccination programs; by poverty and marginalization; and by people’s access to and experiences of vaccination services and frontline healthcare professionals [20]. What we therefore found was that there is a lot about people’s views and practices around vaccination that are not intrinsically about vaccines themselves. Rather, vaccination is often influenced by the relationships people have with institutions, systems, and authorities, by structural conditions and processes, and by social norms and values. However, these more social and political drivers of vaccination still tend to be sidelined within understandings of, and responses to, vaccine hesitancy [2,32]. This is most certainly not a new argument—social scientists undertaking qualitative health research have for some time now been emphasizing the need to better recognize and incorporate the socially situated nature of vaccine decision-making [31,33,34,35]. However, more individualistic and biomedical approaches continue to dominate vaccine hesitancy research and interventions to address it [31,36]. One potential reason for this is that understanding the more macro drivers of vaccine hesitancy often requires in-depth qualitative research that takes time, and tackling these drivers are unlikely to translate into one-dimensional, decontextualized, and ‘quick fix’ strategies. However, decision-making by policy-makers and assessments by donor agencies continue to demand simple and reproducible interventions that are based on ‘big’, quantitative data. These demands pose several limitations. However, if we hope to effectively understand vaccine hesitancy, prioritizing more in-depth qualitative research approaches is essential and urgent. So too is the need to prioritize more multi-faceted and complex strategies that target the social determinants of vaccine hesitancy, along with the provision of education and risk communication.

### 2.3. Vaccine Hesitancy May Be Many Things, Rather Than a Single Phenomenon

Our research has revealed that vaccine hesitancy—the way it manifests and why it occurs—varies considerably across places, populations, time, and even vaccines. That is, we have observed that local contexts and framings matter greatly. For example, in our global and regional systematic reviews, we found that hesitancy towards HPV vaccination was uniquely connected to sociocultural norms surrounding adolescence, sexuality, and gender, and the values people attach to different sexual practices and sexualities [21,24]. Relatedly, it emerged from our research globally [22] and in South Africa [28] that COVID-19 vaccine hesitancy was often driven by concerns about the novelty of the vaccine and speed at which it was developed, which are concerns that did not emerge in the case of more routine vaccines that have been around for many years. Similarly, in our global systematic review, we found that the drivers of hesitancy towards routine childhood vaccines were potentially different for parents from higher and lower resource settings [20]. That is, our review revealed that a worldview informed by neoliberal discourses may be a significant driver of vaccine hesitancy for parents from higher resource settings, whereas for many parents from lower resource settings, their experiences of social exclusion may be a major contributor towards vaccine hesitancy. 

The point is that vaccine hesitancy may not be a single phenomenon, which is something that is widely recognized in the literature [8,37]. However, for the most part, vaccine hesitancy still tends to be spoken about, and intervened upon, in ways that insufficiently appreciate this diversity [36]. We, therefore, need to problematize the ease with which we talk about vaccine hesitancy as a *phenomenon* and potentially move towards conceptualizing the *phenomena* of vaccine *hesitancies* in all their multiplicity. At the same time, the diverse nature of vaccine hesitancy means that single and one-size-fits-all strategies are unlikely to have much traction. Rather, there is a need for multi-component interventions that are tailored to local socio-political contexts, and which target the specific reasons driving vaccine hesitancy in those contexts.

### 2.4. Vaccine Hesitancy May Be an Ongoing ‘Process’ Rather Than a Fixed ‘Stance’

Another common assumption in the vaccine hesitancy literature is that vaccine decisions comprise a more or less fixed position or stance that individuals arrive at in a linear way and at a discrete point in time [33,38,39,40]. However, our [20,21] and others’ [33,38,39,40] research has consistently demonstrated that vaccination decisions may be better understood as dynamic, fluid, and often ambivalent processes. That is, people’s views about vaccination are frequently developed through ongoing interacting and relating, and as such are so often characterized by indeterminacy and an ever-present potential for redevelopment. Importantly, what this procedural nature of vaccination means is that sentiments about vaccines do and potentially can change. This in turn opens up important opportunities—but at the same time challenges—for tracking and responding to vaccine hesitancy, which are issues that we believe require further consideration. 

### 2.5. Vaccine Hesitancy May Sometimes Be about a ‘Striving’ Rather Than a ‘Resisting’

People who are hesitant towards vaccines are often portrayed as ‘resistant’ or ‘selfish’ or even ‘evil’, along with various other stigmatizing labels [34,39,41]. Such constructions have in turn made it challenging for people with differing vaccination views to have civil conversations and have often pushed people to double down on their vaccine hesitancies [35]. One potential way out of this impasse is to appreciate the often positive motivations underpinning people’s concerns about vaccines, as Melissa Leach and James Fairhead demonstrated in their seminal work on vaccine hesitancy over a decade ago [34]. As these authors argue, vaccine anxieties are not inevitably ‘resistance’ in a negative sense but may “also have a more positive sense of an earnest desire *for* something: to strive for improved immunity, strength or resilience, however conceived” [34]. Like Leach and Fairhead and others [34,35,39,41], our research has similarly revealed different ways in which vaccine concerns may be borne out of a ‘striving’ or ‘desiring’ for something, rather than a resisting. 

For example, in our global and regional systematic reviews on routine childhood vaccination, we found that in some instances vaccine hesitancy was about a desire to belong and feel included among peers—a “positively prosocial act” [35] to build social relations and kinship [20,23]. In both of these reviews and our global review on HPV vaccination acceptance, we found that in many cases vaccine hesitancy may be driven by an aspiration to do what’s best for one’s child, albeit based on understandings of well-being that may not align with biomedical understandings of health and immunity [20,21,23]. Sometimes, particularly in contexts of poverty and marginalization, we found that vaccine hesitancy may be a plea for one’s own priorities to be recognized and basic needs met. This was clearly revealed in our global systematic review on routine childhood vaccination and study exploring hesitancy for COVID-19 vaccines in South Africa [20,28]. In a number of our global systematic reviews, we found that vaccine hesitancy may be a yearning for better treatment from healthcare professionals; for a relationship with healthcare professionals in which one feels supported and heard, and in which one’s views are respected and questions answered [20,21,23]. In other situations, and as materialized in many of our studies, vaccine hesitancy may be about a longing for communication that is honest about vaccine risks and uncertainties and transparent about the research and policy- and decision-making surrounding vaccines [20,23,28].

What we and others [34,35,39,41] are therefore suggesting is that in many instances, people’s hesitancies towards vaccination may be underpinned by positive intentions rather than always having negative connotations as is commonly depicted. Importantly, when one recognizes and frames vaccine hesitancies in this way, it opens up potential avenues for public health interventions that are more compassionate and nuanced, and which find ways to work together for common goods [34]. Ultimately, this may help to more effectively and sensitively bridge the goals of vaccination programs with the aspirations of those who have hesitancies towards vaccination [20]. 

### 2.6. ‘Distrust’ as a Driver of Vaccine Hesitancy Needs to Be Better Contextualized and Disaggregated

Distrust has become a pervasive concept in the literature for understanding vaccine hesitancy and what drives it [37,42]. Like others, we have found that a major driver of vaccine hesitancy is people’s distrust in the experts, institutions, or systems implicated with vaccination, including for example scientists, the government, the state-run healthcare system, and the pharmaceutical industry [20,21,22,28]. This distrust emerged in our research as a significant determinant of vaccine hesitancy for various vaccines, amongst numerous populations and within many settings. Importantly, however, what was also revealed was the highly variable and context-dependent nature of distrust, the reasons for it, and how it impacts vaccination views and practices. 

For example, in South Africa we found vaccine hesitancy towards COVID-19 vaccines was intimately connected to a break-down of trust in authorities [28]. Importantly, this distrust emerged as being linked to certain unique dynamics surrounding the pandemic in the country: the corruption scandals, the sometimes ill-conceived and inequitable bans, and the complex geopolitics that created vast vaccine inequities and served as reminders of colonial medical research abuses and more recent patent laws denying local communities access to antiretroviral treatment for HIV/AIDS. A further illustration of the situatedness of distrust is the 2003–2004 polio vaccine boycott in northern Nigeria. Here our research [20] revealed how distrust of government programs and their impact on vaccination was embedded in a complex interplay of local processes and relationships: years of disproportionate poverty and inadequate public services in affected states, religious and political tensions between the northern and southern regions of the country, the infamous Trovan trial in Kano State, as well as the multitude of recently implemented top-down global public health initiatives in the region [20]. HPV vaccination hesitancy in Romania is yet another example of the contextualized nature of distrust, its drivers, and its impact on vaccination. Here our research [21] revealed how many parents’ resistance towards HPV vaccination for their adolescents was entangled with Romania’s complex cultural and historical context of reproductive and sexual exploitation. That is, the country’s history of oppressive, state-driven pro-natalist policies and practices—the legal prohibition of abortion and contraception and women being submitted to mandatory gynecological check-ups at their workplace—had significantly undermined trust in medical professionals and government authorities. Against this backdrop, many women avoided state-run sexual and reproductive healthcare, including HPV vaccination, which was often perceived as yet another form of state exploitation of women’s bodies. 

Therefore, and in sum, we have found that distrust, its drivers, and its impact on vaccination can only be properly understood when situated within the intricacies of particular political events, relations, and processes within particular times and places [20]. However, the notion of distrust within the vaccine hesitancy literature tends to be used in highly generalized and aggregated ways, which arguably obscures more than it reveals. That is, it has become an almost catch-all concept with a self-evident quality, seemingly requiring little further explanation or unpacking [33,34,43]. 

As such, there is a dire need to move away from the “shorthand, universalizing qualities” of the concept of distrust, to explaining why trusting relations between parents and institutions in all their “rich diversity and texture” might break down [34]. This is essential if we hope to develop more meaningful understandings of the role of distrust as a driver of vaccine hesitancy. This is also essential if we hope to develop more effective interventions to (re)build public trust; one’s that are appropriately tailored and targeted to the specific reasons for distrust.

### 2.7. The ‘Demand-Side’ Versus ‘Supply/Access-Side’ Distinction of the Drivers of Suboptimal Vaccination May Be Misleading and Unhelpful

The distinction between ‘supply/access-side’ and ‘demand-side’ issues is commonly made in the literature as a way to understand the reasons for suboptimal vaccination [13,44,45]. Here ‘supply/access-side’ factors are understood to be related to the provision of vaccines and vaccination services, such as the availability and accessibility of effective vaccines, adequate health systems to support their delivery, and health personnel to administer the vaccines. ‘Demand-side’ factors are conceived as those relating to the recipients of vaccines and vaccination services, such as service-users’ knowledge, understanding, attitudes, beliefs, intentions, decision-making, and behaviors. The ‘supply/access-side’ and ‘demand-side’ issues are often conceptualized as distinct from each other [13,44,45], as evidenced, for example, by the WHO’s original definition of vaccine hesitancy as the “delay in acceptance or refusal of vaccination despite availability of vaccination services” [37]. 

Our research has, however, revealed that the so-called ‘supply/access-related’ and ‘demand-related’ dimensions of vaccination are deeply intertwined and often interact in complex ways [20,21,22,28]. For example, across all our studies, we have found that socioeconomic challenges in accessing vaccination services, such as practical questions of geography and transport, competing priorities, and family economics and household work pressures, often contribute to vaccine hesitancy due to the time, effort, and opportunity costs that accessing vaccination involves. Similarly, in our global systematic reviews on acceptance of routine childhood vaccination and HPV vaccination for adolescents, we have found that vaccine hesitancy may be driven by undesirable features of vaccination services and delivery logistics, including, for example, high vaccine costs, vaccine stock-outs and long waiting times at vaccination services [20,21]. The interactions or personal relations people have with frontline healthcare workers, and whether these are experienced as supportive (or not), were also revealed in both these reviews to influence many people’s acceptance of vaccination. 

This intrinsic interconnectedness we have found between the supposed ‘supply/access-side’ and ‘demand-side’ of vaccination suggests that it is potentially misleading and unhelpful to conceptualize and address them separately. There have been recent positive trends in the vaccine hesitancy literature towards collapsing this distinction. For example, understandings of vaccine hesitancy are increasingly incorporating concepts such as “convenience” [46,47] and “practical issues” [48], and conceptual models are shifting to unpacking “behavioral and social drivers of vaccination” [48] and “immunization journey frameworks” [49,50] that seek to integrate so-called ‘supply/access-side’ and ‘demand-side’ issues. The WHO has also recently revised its definition of vaccine hesitancy and now conceives vaccine hesitancy as a “motivational state of being conflicted about, or opposed to, getting vaccinated” [51]. We believe that going forward more research to better understand, and interventions to address, the interplay between different vaccination dimensions is needed. 

## 3. Conclusions

Five years ago, we reflected upon the knowledge advances and gaps on vaccine hesitancy [30,52]. This current reflective and critical commentary piece reiterates many of the issues we raised then, revises some, and suggests certain new issues we think require increased attention going forward. Our hope is that we might see further engagement on these issues and in turn the cultivation of a more critical public health understanding of vaccine hesitancy. In our previous reflections, we suggested that there is a need to incorporate more social science research within the field of vaccine hesitancy, an area that has generally been dominated by biomedical thinking. Today, we are even more convinced of this. The insights we have gained over the last decade, and reflected upon in this commentary piece, have emerged from studies that have utilized critical social science theory and qualitative research methods. These approaches have helped us to acquire in-depth knowledge about the complexities of contexts, processes, relationships, and decision-making dynamics. They have enabled us to unearth and explain vital, but often hard-to-measure, components such as power, politics, and social norms. Ultimately, it is because of these critical social science and qualitative research methodologies that we have been able to better understand, and make recommendations for responding to, the complex phenomenon of vaccine hesitancy. As vaccine hesitancy trends continue to escalate and become even more complicated [53], the need for more critical social science perspectives cannot be overstated. We end our reflections with the famous words of 19th century German physician, Rudolph Virchow, arguably the father of public health:

“Medicine is a social science and politics is nothing else but medicine on a large scale. Medicine as a social science, as the science of human beings, has the obligation to point out problems and to attempt their theoretical solution; the politician, the practical anthropologist, must find the means for their actual solution”.

[54]

## Data Availability

The data presented in this study are all available in the publications cited.
